# A System of Syphilis

**Published:** 1909-09

**Authors:** 


**A System of Syphilis.** Edited by D'Arcy Power, M.B., F.R.C.S., and J. Keogh Murphy, M.D., M.C., F.R.C.S. Vol. II. Pp. viii, 387. London: Oxford Medical Publications. 1908.

In reviewing the first volume of this *System* we expressed the opinion that the work would not suffer by a little judicious compression. Far from retracting that criticism, we are tempted to apply it more forcibly to the second volume.

This bulky tome, in addition to numerous admirable illustrations, contains three sections, devoted to "The Surgery of Syphilis," "The Treatment of Syphilis," and "Syphilis in Obstetrics." With the last named there is little fault to find. Dr. Gow approaches his subject directly, writes of it succinctly, and in merciful remembrance that the careful reader has already given his best attention to the history of syphilis and its pathogeny in the previous volume. He is further to be congratulated on resisting the temptation to indulge in philosophic speculation on the subject of conceptional syphilis (indirect infection of the mother through the fœtus). He is content to lead us to the brink of the inferno with Virgil's admonition to Dante: "Look, and pass on."

Of the effects of syphilis on the ovum Dr. Gow writes lucidly, supporting his own experience with the authoritative views of Fournier. His advice as to the treatment of the pregnant woman who acquires, or has acquired, syphilis is correct and cautious— always treat with mercury during pregnancy when the mother is known to have had syphilis. " There is no doubt of her power to infect the fœtus long after the original attack of syphilis has occurred." " A similar form of treatment will be called for in those cases in which, without any actual history of syphilis, repeated miscarriages, or repeated premature expulsions of a dead fœtus, have occurred."

Criticism of the remainder of the work is rendered difficult by the fact that the " Surgery of Syphilis " is written by one of the editors, Mr. Power, and he must be assumed to be a competent judge of the form this work should take. His acknowledged historical erudition fully entitles him to be heard on the " Early History of Syphilis in Europe," but his very knowledge should have led him to reflect that he had little material information to add to that set forth by Dr. Bloch, and should have made him pause before discoursing further on the pathogenesis of syphilis, where Metchnikoff had already given us of his best. And one further quarrel we have on the score of repetition and inflation : why do we re-discover the title-page of Steber's treatise on syphilis in this volume ?

Mr. Power is in his happiest mood when dealing with the manifestations of syphilis in the bones and joints. Clinical signs and symptoms are interwoven with pathological details in a fashion reminiscent of the golden age of Watson, Latham and Graves. It is hard to have to find fault with any writer who carries the picture in his words, like Mr. Power in his description of syphilitic affections of the mouth or tongue. But we do complain that Mr. Power has yielded to the temptation to repeat the story of Maissoneuve's experimental inoculation of apes, and to refer again to the ill-starred experiment of John Hunter. Volume I contained all this, and more.

Then there is an unnecessary overlapping in the chapters on treatment. A description of the specific treatment of syphilis by ingestion of mercury, inunction and intra-muscular injection, by iodides and by arsenical compounds, would have been welcome either from the pen of Mr. Power or Colonel Lambkin. It is not necessary that two people should say the same thing in almost identical phrases.

It is most interesting to read Colonel Lambkin's experiences in combatting syphilis in the army, where, under adequate discipline and observation, the results of various treatments may be compared. But surely he claims too much for intra-muscular injections of mercury, if he supposes that the mercury is absorbed at an even rate and in regular amount after it has been deposited in the muscles ; for he says : " It is certain that by the intra-muscular method the amount of mercury introduced can be made mathematically correct. Absorption of mercury in a definite manner is ensured by this method." It is such sweeping assertions as these that convince us that even in the medical inspection rooms of our army intra-muscular injection will not hold undisputed sway, still less in private practice.

Chapter xxv, "An Outbreak of Syphilis in a Virgin Soil," is really a section to itself, having no particular reference to the general subject of treatment. Here Colonel Lambkin describes in a graphic manner the introduction of syphilis as a new disease into the Uganda Protectorate, and the ravages it wrought throughout the native community. It is a surprising story, and one which amply corroborates those fifteenth-century chroniclers who relate the incredible spread of syphilis after the Siege of Naples. Colonel Lambkin, in well-weighed words, attributes the outbreak to two chief causes :—

" (1) The introduction of Christianity.

(2) The abolition of the punishments formerly meted out among the tribes for all immoral offences committed by either sex."

We fear that so bald a statement will painfully shock many readers, and it is a serious indictment of missionary effort. But the writer quotes chapter and verse, bringing to his support many well-respected names ; and since in a medical work there is no need for false modesty in language, the conclusion is difficult to combat—that with Christianity is introduced a western idea of equality of the sexes, which to the untutored savage spells only " promiscuous sexual intercourse and immorality." No attack is made on Christian dogma, but rather the fact is made clear that the notion of " emancipated woman " is more eagerly adopted than the restraining principles of a religious morality.

